# Awareness and Knowledge of Amblyopia: A Cross-Sectional Study Among the Population of Hail City, Saudi Arabia

**DOI:** 10.7759/cureus.32194

**Published:** 2022-12-05

**Authors:** Manahel S Almutairi, Njoud S Alanezi, Fatimah A Alshammari, Khulud S Alshammari, Atheer M Alanizy, Arin E Almallahi, Reema S Alanazi, Nabeel M Shalabi, Abrar A Ali

**Affiliations:** 1 Medicine, Hail University, Hail, SAU; 2 Ophthalmology, Hail University, Hail, SAU

**Keywords:** saudi arabia, ksa, hail, knowledge, awareness, amblyopia

## Abstract

Introduction

Amblyopia, also known as the lazy eye, is the reduction of the best-corrected visual acuity of one or both eyes that cannot be attributed exclusively to a structural abnormality of the eye. This study investigated the Hail population’s awareness of amblyopia.

Methods

A cross-sectional study was conducted in Hail, Kingdom of Saudi Arabia (KSA), from May to October 2022. Data entry and analysis were done using RStudio (R version 4.1.1).

Results

This study included 496 of the general population (23.8% males and 76.2% females), and the majority of them had a university degree (65.7%). Of the population, 52.4% knew the definition of amblyopia, 43.4% knew the treatment of amblyopia, and 85.1% knew the importance of checking the child’s vision before school to ensure normal development. In 35.7% of the population, the main source of information was the internet and social media. The median knowledge score of participants was 4 (interquartile range (IQR): 3-5) with a minimum of 0 and a maximum of 9. Based on the univariate analysis, participants aged 41 years or older had significantly lower knowledge scores (β = -0.40, 95% confidence interval (CI): -0.81 to -0.99, p = 0.049), whereas respondents with a positive family history of amblyopia had a significantly higher knowledge score (β = 0.32, 95%CI: 0.02 to 0.61, p = 0.034).

Conclusions

This study assessed the awareness and knowledge of amblyopia among the population in Hail city. According to our data, we found a significantly poor awareness and knowledge compared to other big cities in the same country such as Riyadh and Jeddah. This indicates that knowledge in smaller cities is deficient in enough and accurate sources of knowledge of eye conditions.

## Introduction

Amblyopia, also known as lazy eye, is the reduction of the best-corrected visual acuity of one or both eyes that cannot be attributed exclusively to a structural abnormality of the eye [1]. Amblyopia is one of the most noticeable reasons for visual weakness in the young population [2]. It is a condition that results from abnormal visual development early in life, prompting an absence of excitement in the nerve pathways between the eye and the brain. This makes one eye’s vision weaker, and it gets fewer visual signals [3]. There are several predisposing factors for amblyopia, including refractive error, strabismus that disrupts binocular vision development, and media opacification. Other common causes of amblyopia include early acquired cataracts, vitreous hemorrhage, corneal opacities, and ptosis [1]. Amblyopia can impact the quality of life of children due to its effect on their learning process, their ability to perform physical and social activities, and their career choices [4]. Early diagnosis and treatment are critical to obtaining favorable outcomes in patients with amblyopia [5]. Amblyopia can be improved in the event of early diagnosis and treatment before the age of 9 or 10 [6,7]. In any case, the condition might prompt a visual handicap for the rest of the life if not analyzed and treated accurately [8]. Amblyopia sometimes might associate with various psychiatric issues such as attention deficit hyperactivity disorder (ADHD), hindrances in learning, and dyslexia [9]. This condition can cause permanent effects on the vision of children if not treated early [4] with the appropriate intervention [10]. For that, parents’ understanding of amblyopia and its impact on their children’s well-being is very crucial [11]. The pervasiveness of amblyopia in preschool kids has been recently found to go from 0.8% to 2.6% in populace-based examinations in the United States, Australia, Taiwan, and Singapore [12,13]. The predominance of amblyopia in Saudi Arabia shifts by area: 2.6% in Riyadh [14], 3.9% in Qassim province [10], 1.3% in Jeddah [15], and 1.9% in Abha [16]. These distinctions in commonness could be because of varieties in the definitions and limits of visual boundaries that characterize amblyopia and its attributes. Until now, no study has investigated awareness of amblyopia among the Hail population using a population-based design. Therefore, this study aimed to investigate the Hail population’s awareness of amblyopia.

## Materials and methods

A cross-sectional study was approved by the Research Ethics Committee (REC) of Hail University (reference number: H-2022-28). All participants were informed that the completion of the survey will be considered as approved consent before their enrollment in the study. It was done in June 2022 among the general population of adults > 18 years of age living in Hail province, Saudi Arabia, including urban and rural areas. Data were collected by self-administered electronic Google Forms questionnaire. The researchers created a questionnaire (Appendices) that met our objectives in this study and that was approved by Dr. Abrar Ali Anwar Ali Syed, a professor and consultant at the university medical center of ophthalmology and the Research Ethics Committee (REC) of Hail University.

The study’s sample size of 494 was calculated as 300 participants with a 95% confidence level and 5% margin of error. The calculation was made using the Raosoft sample size software (Raosoft, Inc., Seattle, WA, USA). We collected 496 responses, and data sheets were transferred into an Excel sheet (Microsoft Corp., Redmond, WA, USA) for interpretation. Statical analysis was carried out using RStudio (R version 4.1.1).

Data were expressed as frequencies and percentages (categorical variables) or median and interquartile range (IQR) (continuous variables). Factors associated with the knowledge score were investigated using the linear regression analysis. Univariate models were constructed using the knowledge score as a dependent variable and demographic characteristics as independent variables (each variable in a separate model). Subsequently, significantly associated variables in the univariate analysis were incorporated into a multivariate linear model. Outcomes are expressed as beta coefficients (β) and their respective 95% confidence intervals (95%CIs). Statistical significance was considered at p < 0.05.

## Results

Demographic characteristics of the participants

We received the responses of 496 participants obtained from the online platform. The majority of respondents were females (76.2%) and were residing in the urban region of Hail (86.1%). More than half of them were single (58.9%) and aged 18-25 years (54.4%). Approximately two-thirds of the participants had a university degree (65.7%). A family history of amblyopia was positive among 40.5% of the participants (Table 1).

**Table 1:** Demographic characteristics of the participants

Parameter	Category	Number (%)
Age (years)	18- 25	270 (54.4%)
	26- 40	142 (28.6%)
	41 and older	84 (16.9%)
Gender	Male	118 (23.8%)
	Female	378 (76.2%)
Marital status	Single	292 (58.9%)
	Married	204 (41.1%)
Educational level	Illiterate	6 (1.2%)
	Secondary school or lower	132 (26.6%)
	University	326 (65.7%)
	Postgraduate	32 (6.5%)
Region of residence	Rural regions in Hail	69 (13.9%)
	Urban regions in Hail	427 (86.1%)
Family history of amblyopia	Positive	201 (40.5%)
	Negative	295 (59.5%)

Awareness of amblyopia and its diagnosis

**Figure 1:**
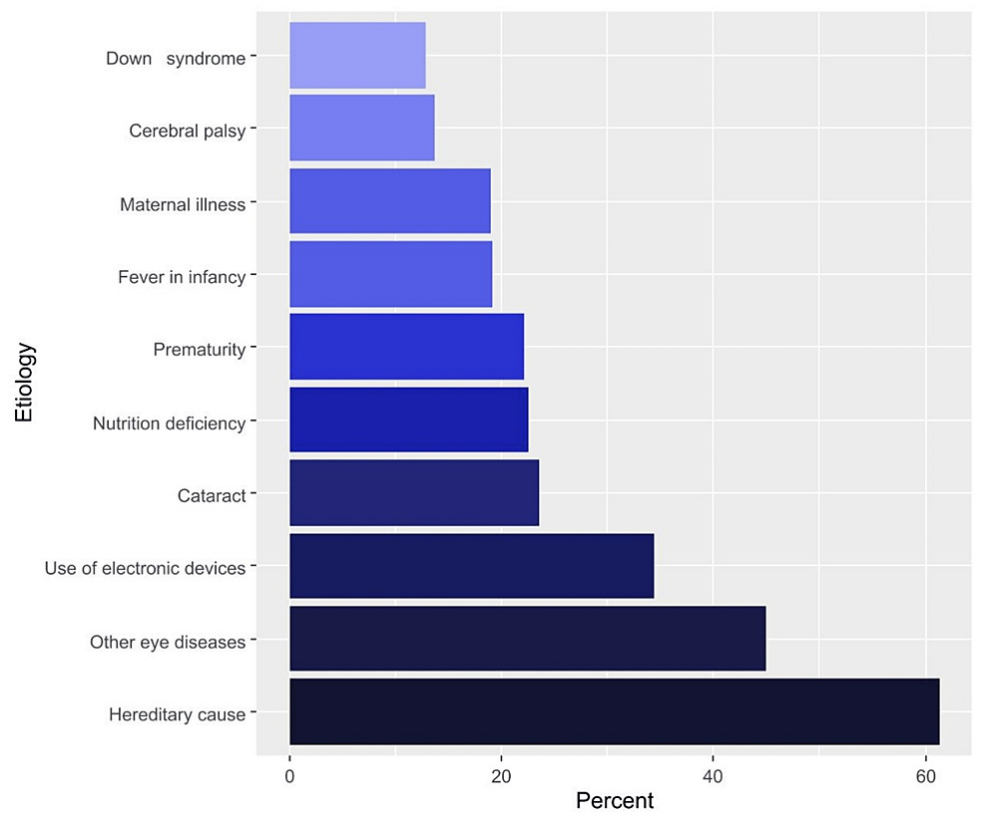
Percentages of participants’ responses regarding their knowledge of amblyopia etiologies

In general, almost half of the respondents had ever heard about amblyopia (53.4%). The most commonly correct answers were related to the fact that amblyopia can be diagnosed by an eye specialist (85.5%) and the importance of checking a child’s vision before school to ensure normal development (85.1%). Approximately one-third of the respondents had correctly indicated that amblyopia is a disease in children (35.7%) and disagreed that a general pediatrician or a family physician can diagnose amblyopia (32.5%). Only 28.2% of the participants correctly answered that amblyopia cannot be detected by the naked eye, and 26.6% of them had correctly perceived that the best age for the treatment of amblyopia is between three and nine years (Table 2). The most frequently perceived etiologies of amblyopia were hereditary reasons (61.3%), other eye diseases (45.0%), and the use of electronic devices (34.5%) (Figure 1). Concerning amblyopia treatment, the most common correct responses were relevant to the application of a patch on the healthy eye (21.8%) and using glasses (21.6%). Of note, the internet and social media (35.7%) and relatives and friends (22.2%) were the most common sources of knowledge about amblyopia, whereas awareness campaigns contributed to knowledge among 11.1% of the participants (Table 3).

**Table 2:** Awareness of amblyopia and its diagnosis An asterisk (*) indicates the correct answer.

Parameter	Category	Number (%)
Have you ever heard of amblyopia?	No	198 (39.9%)
	Yes	265 (53.4%)
	I don’t know	33 (6.7%)
Amblyopia can be detected by the naked eye.	No*	140 (28.2%)
	Yes	135 (27.2%)
	I don’t know	221 (44.6%)
Amblyopia can be diagnosed by a general pediatric or family doctor.	Disagree*	161 (32.5%)
	Agree	174 (35.1%)
	I don’t know	161 (32.5%)
Amblyopia can only be diagnosed by an eye specialist.	Disagree	26 (5.2%)
	Agree*	424 (85.5%)
	I don’t know	46 (9.3%)
Amblyopia is a disease in?	Children*	177 (35.7%)
	Adults	27 (5.4%)
	Both	292 (58.9%)
What is the best age period for the treatment of amblyopia?	Before the age of a year	81 (16.3%)
	Between three and nine years*	132 (26.6%)
	After the age of 10 years	47 (9.5%)
	There is no specific age period	236 (47.6%)
Is it important to check a child’s vision before school for normal development?	No	39 (7.9%)
	Yes*	422 (85.1%)
	I don’t know	35 (7.1%)

**Table 3:** Factors associated with knowledge regarding amblyopia 95%CI: 95% confidence interval, Ref: reference

Parameter	Category	Univariate			Multivariate			
		Beta	95%CI	p	Beta	95%CI	p	
Age	18-25	Ref	Ref		Ref	Ref		
	26-40	-0.14	-0.47, 0.20	0.416	-0.14	-0.47, 0.20	0.417	
	41 and older	-0.40	-0.81, -0.99	0.049	-0.39	-0.80, 0.01	0.054	
Gender	Male	Ref	Ref					
	Female	0.19	-0.15, 0.53	0.269	NA	NA	NA	
Marital status	Single	Ref	Ref					
	Married	0.08	-0.21, 0.38	0.591	NA	NA	NA	
Educational level	Illiterate	Ref	Ref					
	Secondary school or lower	0.45	-0.90, 1.79	0.513	NA	NA	NA	
	University	0.02	-1.30, 1.35	0.975	NA	NA	NA	
	Postgraduate	-0.06	-1.49, 1.37	0.932	NA	NA	NA	
Region of residence	Rural regions	Ref	Ref					
	Urban regions	0.13	-0.29, 0.55	0.533	NA	NA	NA	
Family history of amblyopia	No	Ref	Ref		Ref	Ref		
	Yes	0.32	0.02, 0.61	0.034	0.31	0.02, 0.61	0.037	

The knowledge score and factors associated with amblyopia knowledge

The knowledge score was based on the correct responses to 12 items, including six correct items related to participants’ awareness of amblyopia and its diagnosis, three correct items about amblyopia definition, one correct item about etiology, and two correct items about amblyopia treatment. Therefore, the range of the total score was between 0 and 12. The median knowledge score of participants was 4 (IQR: 3-5) with a minimum of 0 and a maximum of 9. Based on the univariate analysis, participants aged 41 years or older had significantly lower knowledge scores (β = -0.40, 95%CI: -0.81 to -0.99, p = 0.049), whereas respondents with a positive family history of amblyopia had a significantly higher knowledge score (β = 0.32, 95%CI: 0.02 to 0.61, p = 0.034). However, on the multivariate analysis, only having a family history of amblyopia was a significant independent predictor of higher knowledge scores (β = 0.31, 95%CI: 0.02 to 0.61, p = 0.037) (Table 4).

**Table 4:** Factors associated with knowledge regarding amblyopia 95%CI: 95% confidence interval, Ref: reference

Parameter	Category	Univariate			Multivariate			
		Beta	95%CI	p	Beta	95%CI	p	
Age	18- 25	Ref	Ref		Ref	Ref		
	26-40	-0.14	-0.47, 0.20	0.416	-0.14	-0.47, 0.20	0.417	
	41 and older	-0.40	-0.81, -0.99	0.049	-0.39	-0.80, 0.01	0.054	
Gender	Male	Ref	Ref					
	Female	0.19	-0.15, 0.53	0.269	NA	NA	NA	
Marital status	Single	Ref	Ref					
	Married	0.08	-0.21, 0.38	0.591	NA	NA	NA	
Educational level	Illiterate	Ref	Ref					
	Secondary school or lower	0.45	-0.90, 1.79	0.513	NA	NA	NA	
	University	0.02	-1.30, 1.35	0.975	NA	NA	NA	
	Postgraduate	-0.06	-1.49, 1.37	0.932	NA	NA	NA	
Region of residence	Rural regions	Ref	Ref					
	Urban regions	0.13	-0.29, 0.55	0.533	NA	NA	NA	
Family history of amblyopia	No	Ref	Ref		Ref	Ref		
	Yes	0.32	0.02, 0.61	0.034	0.31	0.02, 0.61	0.037	

## Discussion

In this study, we aimed to assess the awareness and knowledge of amblyopia among the population in Hail city because amblyopia is a common cause of pediatric visual impairment and can permanently affect vision if not treated early and therefore can impact health and quality of life.

Awareness of amblyopia and its diagnosis

Our research outcome on awareness, detecting, and diagnosing amblyopia in the general population is low. There is a large difference in the participants’ responses regarding whether general practitioners (GPs) or family physicians can diagnose amblyopia. The vast majority agree or not knowing. In contrast, the majority agree that amblyopia is diagnosed by an eye specialist. Moreover, less than half of the participants are aware that children are exposed to amblyopia instead of adults. These responses appear similar to the study conducted among pediatric and ophthalmology clinics at King Abdulaziz University Hospital in Jeddah [17] and King Abdullah Specialist Children Hospital in Riyadh [18]. We think that these similarities are because of the fact that the study was conducted in the same country, and there is a lack of awareness programs for amblyopia. Unfortunately, half of the participants believe that there is no specific age period to treat amblyopia, and this is unfortunate because it must be treated very early or it may lead to permanently affected vision, in contrast to the study conducted in Jeddah [17] and Riyadh [18], which showed a good knowledge of the importance of treating amblyopia early and at a young age.

Knowledge regarding amblyopia

Based on the various age groups and levels of education that participants in this study represent, we wanted to know how well they understood the correct description of amblyopia. Only 6.2% of the respondents correctly identified that amblyopia may result in a visual loss in one eye. Of the respondents, 23.6% said that amblyopia is caused by a problem between the eyes and the brain not working well together, and 22.6% of the participants believed that amblyopia is caused by decreased vision in one or both eyes. Other participants misidentified amblyopia as eyes that do not line up in the same direction (11.9%), degeneration of the optic nerve (11.1%), abnormal eye movement (8.1%), decreased night vision (2.6%), and the inability of the eye to move (13.9%). The 2019 study by Alsaqr et al. found that 30% of the participants were knowledgeable about the disease [5]. According to Alzahrani et al., 49.7% of the participants were also knowledgeable about the disease and its etiology [17], which is higher than our study and that of Alsaqr et al. [5]. This may be accounted for by the fact that the study sample comprised patients who attended pediatric and ophthalmology clinics at a tertiary hospital, where parents with medical knowledge are more likely to be present. Consistent with our data, the level of amblyopia knowledge is more significant than that reported in Nigeria (2.9%) [19] and India (3%) [20].

The knowledge score and factors associated with amblyopia knowledge

Regarding amblyopia etiologies in our research, there is only one answer the participants got correctly, which is cataract, and none have chosen refractive error as an important reason. On the other hand, the majority chose hereditary and genetics as the leading cause. In contrast, a previous study in Jeddah found that most of the responders have chosen strabismus and refractive errors [5]. By observation, there are variations in knowledge level among parents in Jeddah and Hail with a significantly higher level in Jeddah, and we believe that our outcome reflects more realistic results as the previous study conducted the data among parents attending the amblyopia awareness campaign. Amblyopia treatment results are considered satisfying as the majority of responders answered that an eye patch in the normal eye and glasses are the primary treatment, and the minority respond that it will resolve spontaneously, similar to the same study from Jeddah. We do think preschool eye checkup is a significant source of society’s knowledge about treatment, as it has been demonstrated in our outcome in the parents’ awareness of amblyopia as the majority agreed about preschool examination for children what considered a reassuring sign. Primary sources of information about amblyopia in the current study were the internet, social media, friends, and family. In contrast, in the study by Alsaqr et al., the primary sources were physicians and the internet [5]. This may be considered a factor behind the inaccurate answers in our study. Overall, the awareness and knowledge of amblyopia among the population in Hail city are considered weak and need more attention through seminars, campaigns, or intensive programs about amblyopia, since it is a problem that is not uncommon, to overcome future preventable consequences.

Limitations

This was the first study in Hail to assess the awareness and knowledge of amblyopia among the Hail population. There were some possible limitations in this study, such that the yes-no questions in the survey (numbers 8, 9, and 11) might influence the participant to choose the correct answers. Furthermore, there was no financial support for the study, which we also considered an obstacle.

## Conclusions

This study assessed the awareness and knowledge of amblyopia among the population in Hail city, and we found a significantly poor and weak awareness and knowledge compared to other big cities such as Riyadh and Jeddah. This indicates that knowledge in smaller cities is deficient in enough and accurate sources of knowledge about eye conditions.

Since we found that the awareness and knowledge of amblyopia among the population in Hail city are considered weak compared to other large cities, we recommend future researchers to conduct surveys manually instead of online questionnaires for a more accurate result. Furthermore, the Hail population needs more attention through seminars, campaigns, media commercials, and intensive programs about amblyopia. In the long run, a small city such as Hail with a large population needs a hospital that specializes in tertiary eye care specifically.

## Appendices

Assessment of the awareness and knowledge of amblyopia among the population of Hail city

We are a group of students from the Faculty of Medicine at the University of Hail, and we will conduct our scientific research entitled “Assessment of Awareness and Knowledge of Amblyopia Among the Population of Hail City.”

We kindly ask you to fill out the questionnaire. It does not take more than two minutes, and it does not require any information that identifies the person, such as name and phone number. The required data will be used for scientific research purposes only and will be used in complete confidentiality.

Thank you for your cooperation.

Your completion of this questionnaire is considered consent to participate in the research.

Questionnaire

1. Age

○     18-25

○     26-40

○     41 and older

2. Gender 

○     Female

○     Male

3. Marital status

○     Single

○     Married

4. Family history of eye disease

○     Yes

○     No

5. Educational level

○     High school or less

○     College degree

○     Postgraduate

○     Illiterate

6. Have you ever heard of amblyopia?

○     Yes

○     No

○     I don’t know.

7. Amblyopia can be detected by the naked eye.

○     Yes

○     No*

○     I don’t know.

8. Amblyopia can be diagnosed by a general pediatric or family doctor.

○     Yes

○     No*

○     I don’t know.

9. Amblyopia can only be diagnosed by an eye specialist.

○     Yes*

○     No

○     I don’t know.

10. Amblyopia is a disease in?

○     Children*

○     Adult

○     Both

11. Is it important to check a child’s vision before school for normal development?

○     Yes*

○     No

○     I don’t know.

12. What is the best age period for the treatment of amblyopia?

○     After the age of 10 years

○     Before the age of a year

○     Between three and nine years*

○     There is no specific age period.

13. Amblyopia definition

○     The eye and brain not working well together*

○     Two eyes don’t line up in the same direction

○     Degeneration of the optic nerve

○     Decreased vision in one or both eyes*

○     Abnormal eye movement

○     Decreased night vision

○     Vision loss in one eye*

○     Misalignment of both eyes

○     Inability of the eye to move

○     Misalignment of an eye

14. Amblyopia etiologies (you can choose more than one)

○     Maternal illness

○     Prematurity*

○     Fever in infancy

○     Electronic device use

○     Cerebral palsy

○     Down syndrome*

○     Nutrition deficiency

○     Cataract*

○     Trauma

○     Hereditary cause

○     Refractive error*

15. Knowledge about amblyopia treatment

○     Resolves spontaneously

○     Laser therapy

○     Patch on the healthy eye*

○     Eye muscle exercise

○     Glasses*

○     Surgery is the best treatment

16. Sources of knowledge about amblyopia

○     Books

○     Awareness campaigns

○     Doctor

○     Internet/social media

○     Relatives/friends
